# Epidemiological characteristics and trends of notified enteric fevers in Germany, 2001 to 2023

**DOI:** 10.2807/1560-7917.ES.2025.30.14.2400314

**Published:** 2025-04-10

**Authors:** Julia Enkelmann, Sandra Simon, Eva Trost, Klaus Stark, Christina Frank

**Affiliations:** 1 Robert Koch Institute, Department of Infectious Disease Epidemiology, Berlin, Germany; 2 Robert Koch Institute, Unit of Enteropathogenic Bacteria and Legionella, National Reference Center for Salmonella and Other Bacterial Enterics, Wernigerode, Germany

**Keywords:** Typhoid fever, paratyphoid fever, enteric fever, *Salmonella* Typhi, *Salmonella* Paratyphi, Germany, surveillance data, antimicrobial resistance

## Abstract

**Background:**

Enteric fevers (EF) are caused by infections with *Salmonella* Typhi (STY) or *Salmonella* Paratyphi (SP) A-C (except the SPB enteric pathovar) and exhibit increasing antimicrobial resistance (AMR). Notification is mandatory in Germany.

**Aim:**

To describe characteristics and trends of notified EF cases in Germany.

**Methods:**

We analysed German EF notifications 2001–2023 fulfilling the case definition. We calculated numbers of imported EF cases per 100,000 air travellers by country of exposure 2012–2023.

**Results:**

In 2001–2023, 2,670 confirmed EF cases were notified: 56% (1,498/2,670) STY, 44% (1,172/2,670) SP, with seasonal peaks in April–May and August–September. Aside from years with COVID-19-related travel restrictions, STY notifications were stable, while SP notifications decreased. Median age of EF cases was 26 years (range: 0–93) and 55% (1,458/2,663) were male. Of cases with information, 93% (2,491/2,670) had fever, 71% (1,906/2,670) diarrhoea, 78% (2,033/2,607) were hospitalised (STY: 85% (1,234/1,459) vs SP: 70% (799/1,148), p < 0.001) and four died (two STY, one SPA, one SPB). Of STY cases, 7% (88/1,221) reported vaccination. Overall, 86% (2,251/2,613) of cases acquired EF abroad, most commonly in India, Pakistan and Türkiye. Ciprofloxacin resistance was reported for 50/59 STY and 16/18 SPA cases and cefotaxime resistance for 10/57 STY cases (exposure: Pakistan (9/10), India (1/10)) with information since 2017. We also report outbreaks and incidence among travellers.

**Conclusions:**

Most cases were imported and had high hospitalisation rates and AMR. Typhoid vaccination was underutilised, highlighting that additional ways to reach at-risk travellers with information and vaccination offers are needed.

**Key public health message**

**What did you want to address in this study and why?**

Typhoid and paratyphoid fever are important travel-related infections. We describe trends and epidemiologic characteristics of notified cases in Germany, 2001–2023, including place of infection, vaccination status and selected antimicrobial resistance (AMR) by organism to inform clinicians and public health experts. To estimate risk of infection from travel, we calculated travel-related cases per 100,000 air travellers by destination, 2012–2023.

**What have we learnt from this study?**

Demographic characteristics, place of infection and trends differed by organism and over time. Estimated incidence per 100,000 air travellers was highest for Pakistan and Bangladesh. Most *Salmonella* Typhi (STY) cases were unvaccinated. Ciprofloxacin resistance was reported for most STY and *Salmonella* Paratyphi A cases with information, while cefotaxime resistance was mainly reported for STY cases returning from Pakistan.

**What are the implications of your findings for public health?**

Our data may support risk-assessment in travel medicine and provide a basis for targeted pre-travel advice and vaccination as well as identifying affected groups. Antimicrobial resistance data by country of acquisition are useful in guiding empirical therapy. Therefore, systematic AMR monitoring of all relevant antibiotics by country of acquisition would be of value and further inform public health strategy.

## Introduction

Typhoid and paratyphoid fever - collectively known as enteric fevers (EF) - are caused by infections with *Salmonella enterica* subspecies *enterica* serovars Typhi (STY), Paratyphi A (SPA), B (SPB, only systemic pathovar) or C (SPC) and are notifiable according to the German Protection against Infection Act [1]. Transmission mainly occurs via ingestion of food including water, contaminated by faeces of infected individuals. With an estimated 14.3 million cases and 135,900 deaths worldwide in 2017 [2], EF remain major public health concerns, mainly affecting countries with poor sanitation and lack of clean drinking water. Endemic regions include Asia (especially South Asia), Africa, Central and South America and parts of Oceania [3].

Enteric fevers are potentially life-threatening and clinically indistinguishable [4], although SPB infection is often considered to cause milder courses of illness. The clinical picture is variable. Typical symptoms include fever, headache and malaise. Both constipation and diarrhoea can occur. The most common complications are intestinal bleeding (up to 10% of hospitalised patients with severe EF), intestinal perforations (up to 3% of hospitalised patients) or neurologic manifestations (2–40%) [4,5].

Early antibiotic therapy can reduce the case fatality rate to less than 1%, compared with up to 20% in the preantibiotic era [4]. However, treatment is becoming more challenging with emerging antimicrobial resistance (AMR). Since 2016, infections with extensively drug-resistant (XDR)-STY have occurred endemically in Pakistan, displaying resistance against chloramphenicol, ampicillin, trimethoprim/sulfamethoxazole, fluoroquinolones and third generation cephalosporins [6]. This emphasises the importance of prevention measures, including sanitation, access to clean water, adherence to hygiene practices and typhoid vaccination.

Typhoid vaccines available in Germany are licensed for individuals 2 years or older. Vaccine efficacy against STY is about 50–70% and the vaccines provide no or very limited protection against *S.* Paratyphi (SP) [7,8]. Vaccination is currently recommended for: (i) all travellers to Afghanistan, Bangladesh, India, Nepal and Pakistan independent of travel style, (ii) travel with basic travel/accommodation/working conditions to all endemic regions with low hygiene standard (e.g. trekking, aid missions), especially during outbreaks and disasters, (iii) stays in endemic regions for longer than 4 weeks, and (iv) people with a migration background travelling to their home country that has increased risk [7].

Conjugate typhoid vaccines - with better efficacy and feasible for children 6 months or older [9] - are currently not licensed or available in Germany.

We aim to describe epidemiological characteristics and trends of notified EF in Germany between 2001 and 2023 and estimate the risk for travellers by country by calculating incidences per 100,000 air-travellers to that destination to inform clinicians and policymakers.

## Methods

### Enteric fever surveillance

In Germany, it is mandatory for laboratories to notify the direct detection of STY or SP and for physicians to notify clinical suspicion, disease or death due to EF (within 24 hours) to the responsible local public health authority, which assesses the case and implements measures as required.

Information (notified and collected during the investigation) on age, sex, district of residence, laboratory method used, date of disease onset and notification, relevant symptoms, hospitalisation, death, vaccination status, place(s) of exposure (with dates if ascertainable) and identified epidemiological links is then electronically transmitted in pseudonymised form to both the federal public health authority and the national health institute (Robert Koch institute (RKI)). Additional relevant information can be included as a comment. Antibiotic susceptibility data are currently not routinely captured within the national surveillance system, but since mandatory notification of Enterobacterales with carbapenem-nonsusceptibility or with evidence of a carbapenemase determinant came into effect in Germany on 1 May 2016 [10], selected additional antibiotic susceptibilities, e.g. to ciprofloxacin (fluoroquinolone) and cefotaxime (a proxy for ceftriaxone; third generation cephalosporin), can be reported voluntarily, which we include in the analysis. No data on macrolides were available.

Submission of isolates to the national reference centre (NRC) for whole genome sequencing with subsequent multi locus sequence (MLST) typing of STY isolates and subtyping of SP isolates is encouraged but is voluntary and the results are not yet automatically linked to the notification software and were not available for analysis.

### Case definition and analysis

Cases are considered confirmed if they fulfil the clinical criteria of a fever (body temperature ≥ 38.5 °C or anamnestic information suggestive of fever) OR two of the three following criteria: (i) diarrhoea, constipation or abdominal pain, (ii) cough, (iii) headache OR death due to EF) AND are laboratory confirmed (culture positive OR, since September 2023, PCR positive). Instead of being laboratory confirmed, a case can be epidemiologically linked with a laboratory-confirmed human infection through human-to-human contact, shared source of exposure (e.g. food) OR consumption of food or water in which STY or SP was detected [11].

We analysed confirmed EF cases notified in Germany between 1 January 2001 and 31 December 2023. Data were retrieved at RKI from the software for the collection of notification data on 2 January 2024. Missing information provided in comments was transferred to the dataset. We excluded 16 SPB infections with comments indicating misclassification of the enteric pathovar (enteric pathovar, Java, tartrate positive), which is notifiable as non-typhoidal salmonellosis and does not cause EF. Cases with SPC were rare and are not presented in detail.

Cases for which local public health authorities provided at least one suspected place of infection abroad were considered imported. For the purpose of this analysis, we considered Türkiye to be a part of Asia, as the majority of the population and landmass is located there and the majority of cases imported from Türkiye with additional location information were exposed in the Asian part of Türkiye. Implausible dates, e.g. symptom onset of imported cases before stay abroad began, return dates before departure and duration of hospital stay less than 0 days were recoded as missing. We present autochthonous outbreaks or international outbreaks related to other nonendemic countries with at least three cases that were not limited to one household. National travel vaccination recommendations were used to evaluate if cases with reported vaccination were current on their typhoid vaccination [7]. Intermediary antibiotic susceptibility as per laboratory report was considered susceptible.

We used population data from the Federal Office of Statistics to calculate incidence per 100,000 population. Stata version 17.0 (StataCorp., College Station, United States (US)) and Microsoft Excel were used to carry out descriptive statistical analysis. We compared categorial variables using the Pearson chi-squared test and ages using the Wilcoxon rank-sum test (Mann Whitney). P values below 0.05 were considered significant.

Regiograph Analyse (GfK GmbH, Nuremberg, Germany) was used to create maps.

### Incidence of imported cases by country

For reported countries of infection with more than five entries as sole place of infection between 2012 and 2023 overall or per serotype (STY, SPA or SPB), we obtained the number of air travellers departing from Germany with last known destination to that country from the air traffic transport performance statistic [12]. This was used to estimate incidence rates per 100,000 air travellers overall and by serotype, in 4-year intervals. Syria was not included in this analysis as the majority of cases imported from Syria during 2012–2023 were people from that country seeking asylum in Germany and it often was unclear where their place of infection had been.

## Results

Overall, 2,670 confirmed EF cases were notified in Germany between 2001 and 2023, corresponding to a mean annual incidence of 1.4 per 1,000,000 population. Of these, 56.1% (1,498/2,670) had STY infection and 43.9% (1,172/2,670) had SP infection.

Of 1,110 SP cases with available pathovar, 50.6% (562/1,110) were infected with SPA, 47.7% (529/1,110) with SPB and 1.7% (19/1,110) with SPC.

The annual number of STY notifications remained similar until 2020, when all EF notifications decreased considerably, coinciding with travel restrictions during the COVID-19 pandemic. The number of SP notifications (especially SPB) had already appeared to decrease over the years prior to 2020. Enteric fever notifications remained low in 2021, increased in 2022 and reached pre-pandemic levels in 2023.

**Figure 1:**
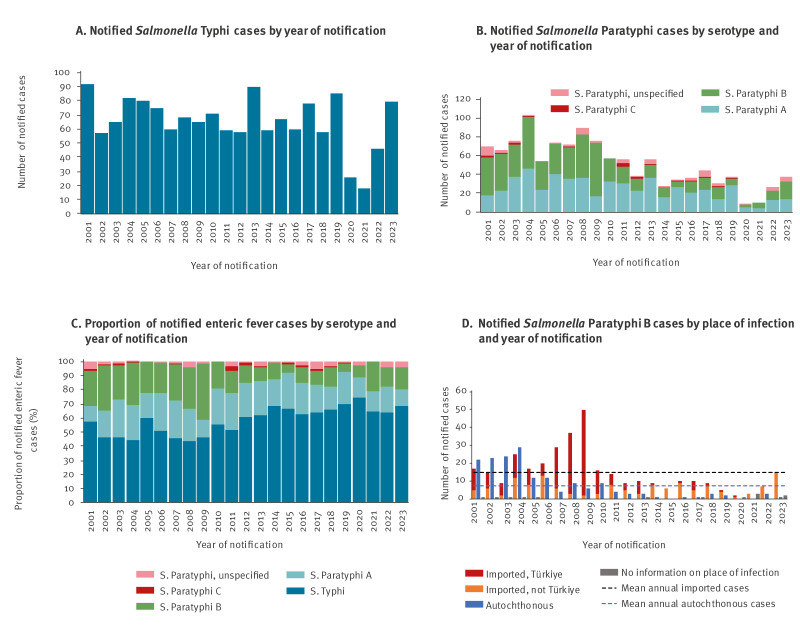
Number of notified A) *Salmonella* Typhi cases by year of notification, and B) *S.* Paratyphi cases by serotype and year of notification, C) proportion of notified enteric fever cases by serotype and year of notification, and D) number of *S.* Paratyphi B cases by place of infection and year of notification, Germany, 2001–2023

Notifications and proportions by serotype and year are shown in Figure 1.

**Figure 2:**
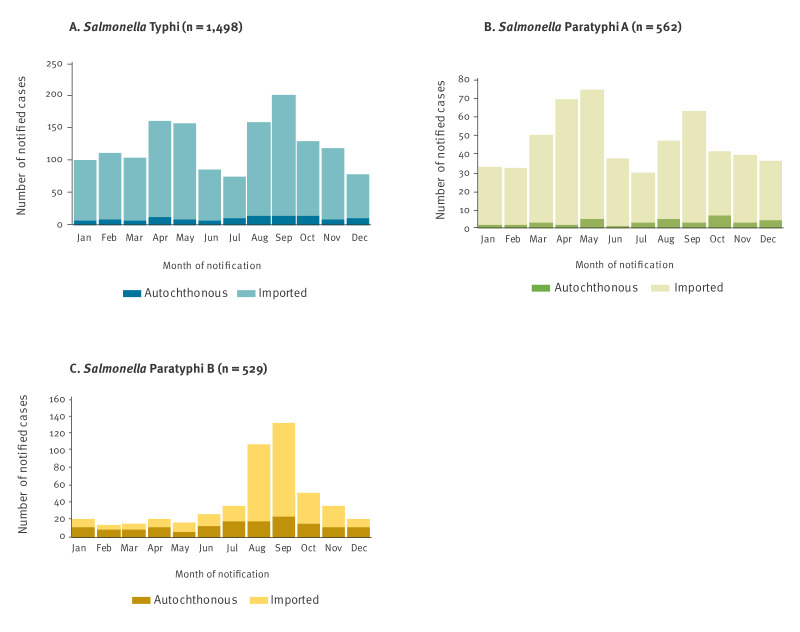
Number of notified cases by month of notification, place of infection and serotype for A) *Salmonella* Typhi (n = 1,498), B) *S.* Paratyphi A (n = 562), C) *S*. Paratyphi B (n = 529), Germany, 2001–2023

There was a seasonal increase in the number of notified STY and SPA infections in April–May and in August–September, while notifications of SPB only peaked in late summer. Autochthonous cases occurred throughout the year, constituting a small minority, except for SPB (Figure 2) before 2013 when 37.8% (157/415) of SPB cases were reported as autochthonous.

### Demographic and clinical information

Enteric fevers occurred in all age groups and 54.8% of cases with information were male. Median age was 26 years (range: 0–93), varying by serotype between 16 years (SPB) and 32 years (SPC). Overall, 30% were younger than 18 years old (SPB: 54.8%, STY: 27.0%, SPA: 14.8% Table 1). Supplement S1 shows demographic, epidemiological and clinical information and suspected country of acquisition of notified cases of enteric fevers in Germany by serotype and time period (2001–2012 and 2013–2023).

**Table 1:** Demographic, epidemiological and clinical information of notified cases of enteric fever with subgroup information for *Salmonella* Typhi, *S.* Paratyphi A and *S.* Paratyphi B^a^ and suspected country of acquisition by serotype, Germany, 2001–2023

Demographic information	EF, total (n = 2,670)		STY (n = 1,498)		SPA (n = 562)		SPB (n = 529)	
	%	n/N^b^	%	n/N^b^	%	n/N^b^	%	n/N^b^
Sex								
Male	54.8	1,458/2,663	55.1	821/1,491	60.1	338/562	51.0	270/529
Female	45.3	1,205/2,663	44.9	670/1,491	39.9	224/562	49.0	259/529
Age								
< 18 years	30.0	800/2,667	27.0	404/1,495	14.8	83/562	54.8	290/529
19–39 years	47.7	1,272/2,667	50.8	759/1,495	56.8	319/562	30.1	159/529
40–59 years	16.6	442/2,667	16.3	243/1,495	23.5	132/562	9.8	52/529
≥60 years	5.7	153/2,667	6.0	89/1,495	5.0	28/562	5.3	28/529
Handling food								
For work	3.4	74/2,183	3.8	45/1,190	4.3	20/467	1.5	7/466
**Autochthonous cases**	**13.9**	**362/2,613**	**8.7**	**8.7**	**7.3**	**40/551**	**33.3**	**172/344**
Sex								
Male	53.0	192/362	54.3	69/127	65.0	26/40	51.7	89/172
Female	47.0	170/362	45.7	58/127	35.0	14/40	48.3	83/172
Age								
< 18 years	45.0	163/362	32.3	41/127	32.5	12/40	59.3	102/172
≥ 60 years	13.0	47/362	15.0	19/127	15.0	6/40	9.3	16/172
**Imported cases**	**86.2**	**2,251/2,613**	**91.4**	**1,341/1,468**	**92.7**	**511/551**	**66.7**	**344/516**
Sex								
Male	55.0	1,234/2,244	55.2	736/1,334	59.7	305/511	50.3	173/344
Female	45.0	1,010/2,244	44.8	598/1,334	40.3	206/511	49.7	171/344
Age								
< 18 years	27.5	618/2,249	26.6	356/1,339	13.5	69/511	52.3	180/344
≥ 60 years	4.5	101/2,249	5.0	67/1,339	4.1	21/511	3.2	11/344
Region of importation								
From Asia (including Türkiye^c^)	83.7	1,869/2,233	81.4	1,080/1,327	97.2	494/508	74.6	256/343
From Africa	6.7	150/2,233	9.7	128/1,327	1.6	8/508	1.8	6/343
From the Americas	6.2	138/2,233	6.4	85/1,327	0.4	2/508	13.1	45/343
From Europe	2.9	64/2,233	1.9	25/1,327	0.6	3/508	9.9	34/343
Exposure in multiple continents	0.5	12/2,233	0.7	9/1,327	0.2	1/508	0.6	2/343
Countries of importation (in order of numbers of cases)								
Top 1 country of acquisition^d^	India: 38.6	824/2,133	India: 42.1	534/1,269	India: 55.2	267/484	Türkiye: 61.5	203/330
Top 2 country/ies of acquisition^d^	Pakistan: 14.2	302/2,133	Pakistan: 17.7	224/1,269	Pakistan: 15.7	76/484	Bolivia: 4.2	14/330
Top 3 country/ies of acquisition^d^	Türkiye: 13.1	280/2,133	Bangladesh: 4.8	61/1,269	Nepal: 3.7	18/484	Peru, Iraq: 3.3	11/330
**If imported: duration of stay abroad** ^e^								
≤ 1 week^e^	4.7	69/1,481	3.4	29/865	6.4	21/330	6.4	16/250
> 1–2 weeks^e^	12.0	178/1,481	10.8	93/865	14.6	48/330	12.0	30/250
> 2–4 weeks^e^	35.9	531/1,481	37.2	322/865	30.6	101/330	37.6	94/250
> 1–2 months^e^	24.2	359/1,481	22.8	197/865	22.7	75/330	32.4	81/250
> 2–6 months^e^	20.7	306/1,481	22.8	197/865	22.7	75/330	11.2	28/250
> 6 months^e^	2.6	38/1,481	3.1	27/865	3.0	10/330	0.4	1/250
**If imported: timing of symptom onset** ^f^								
During stay abroad^f^	30.8	448/1.453	29.0	252/868	26.2	85/324	43.1	97/225
> 0–10 days after stay abroad^f^	44.0	639/1,453	42.1	365/868	44.8	145/324	48.4	109/225
11–28 days after stay abroad^f^	20.4	296/1,453	23.7	206/868	22.5	73/324	6.7	15/225
29–60 days abroad^f^	4.7	68/1,453	5.0	43/868	6.5	21/324	1.8	4/225
> 60 days after stay abroad^f^	0.1	2/1,453	0.2	2/868	0	0/324	0	0/225
**Clinical information**								
Fever	93.3	2,491/2,670	94.9	1,421/1,498	96.6	543/562	86.2	456/529
Diarrhoea	71.4	1,906/2,670	68.6	1,028/1,498	63.4	356/562	85.6	453/529
Constipation	5.2	138/2,670	6.7	100/1,498	3.9	22/562	2.7	14/529
Abdominal pain	35.9	958/2,670	35.5	532/1,498	33.1	186/562	38.8	205/529
Cough	8.2	219/2,670	8.9	133/1,498	11.2	63/562	3.4	18/529
Headache	29.4	785/2,670	31.0	464/1,498	32.9	185/562	21.6	114/529
Severity of disease								
Hospitalised	78.0	2,033/2,607	84.6	1,234/1,459	78.3	429/548	62.9	327/520
Deaths due to notified disease	0.2	4/2,636	0.1	2/1,473	0.2	1/560	0.2	1/523

EF: enteric fevers; SPA: *Salmonella* Paratyphi A; SPB: *S*. Paratyphi B; STY: *S.* Typhi.

^a^ Information by time period (2001–2012 and 2013–2023) is provided in Supplement S1.

Information on *S*. Paratyphi C and unspecified *S.* Paratyphi is not shown in the table.

^b^ Number of cases with information.

^c^ For the purpose of this analysis we considered Türkiye to be a part of Asia, as the majority of the population and landmass is located there and the majority of cases imported from Türkiye with additional location information were exposed in the Asian part of Türkiye.

^d^ If only exposed in one country abroad.

^e^ If information on duration of stay was available, known new entrants or visitors to Germany were excluded.

^f^ If information on timing of symptom onset was available.

**Figure 3:**
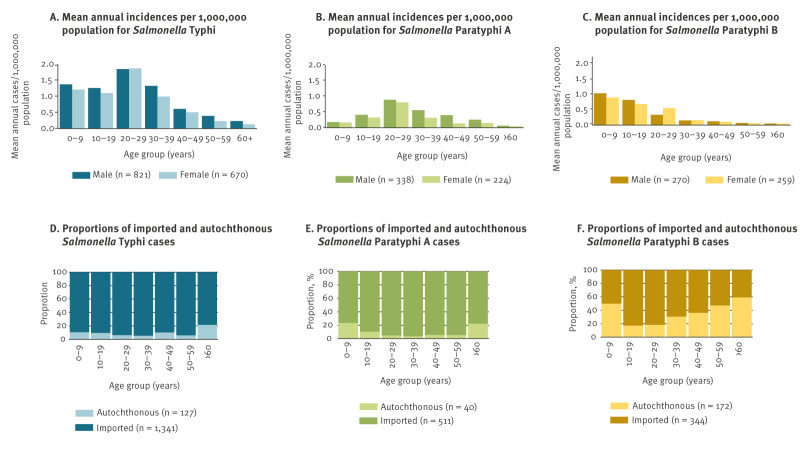
Age- and sex-specific mean annual incidences per 1,000,000 population for A) *Salmonella* Typhi, B) *S*. Paratyphi A, C) *S.* Paratyphi B, and proportions of imported and autochthonous cases by age group and serotype for A) *S*. Typhi, B) *S*. Paratyphi A, C) *S.* Paratyphi B, Germany, 2001–2023

Age- and sex-specific incidences by serotype and proportions of imported vs autochthonous cases by age group are shown in Figure 3.

Fever was the most commonly reported symptom (93.3%), followed by diarrhoea (71.4%), abdominal pain (35.9%), headache (29.4%), cough (8.2%) and constipation (5.2%). Of all cases, 78% (2,033/2,607 with information) were hospitalised for a median of 9 days (duration available for n = 867, range: 0─57 days). *Salmonella* Typhi cases were significantly more likely to be hospitalised than SP cases (84.6% vs 69.6% (799/1,148), p < 0.0001) and SPA cases were significantly more likely to be hospitalised than SPB cases (78.3% vs 62.9%, p < 0.0001) (Table1). Overall, EF admission rates were similar for those under 18 years of age and adults (79.1% (620/784) vs 77.5% (1,411/1,820), p = 0.38), but for SPB they were significantly higher in those under 18 years (69.1% (197/285) vs 55.3% (130/235) in adults, p = 0.001).

Four EF-related deaths were notified, most recently in 2007. The causative serotypes were STY infection in two travellers returning from India (different years, one child and one adult aged 45–60 years), SPA infection in one case with unknown place of infection (adult < 30 years) and SPB infection in one autochthonous case (adult > 60 years). In three of these cases the diagnosis was made post mortem.

### Vaccination

Of 1,221 STY cases with information, 88 (7.2%) reported vaccination against typhoid fever. Of 29 vaccinated cases with sufficient information, 20 cases were considered current on their typhoid vaccination based on national travel vaccination recommendations. There were 26 notified STY cases in children younger than 2 years and not eligible for typhoid vaccines currently licensed in Germany, but no reported deaths.

### Geographical distribution and countries of infection

Cases with EF were reported from all 16 German states. Of 2,613 cases with information, 13.9% (n = 362; SPB: 33.3% (172/516), STY: 8.7% (127/1,468), SPA: 7.3% (40/511)) were autochthonous and 86.2% (n = 2,251) were imported, with median ages of 21 and 26 years, respectively. Compared with imported cases, autochthonous cases were significantly more likely to be under 18 years of age (45.0% (163/362) vs 27.5% (618/2,249), p < 0.0001, by subtype this was only significant for SPA) or 60 years or older (13.0% (47/362) vs 4.5% (101/2,249), p < 0.0001, significant for all subtypes). Of autochthonous cases, 83.7% (303/367) were notified before 2013 and a link to another case or an outbreak was reported for 37 SPB cases, 31 STY cases and one SPA case.

**Figure 4:**
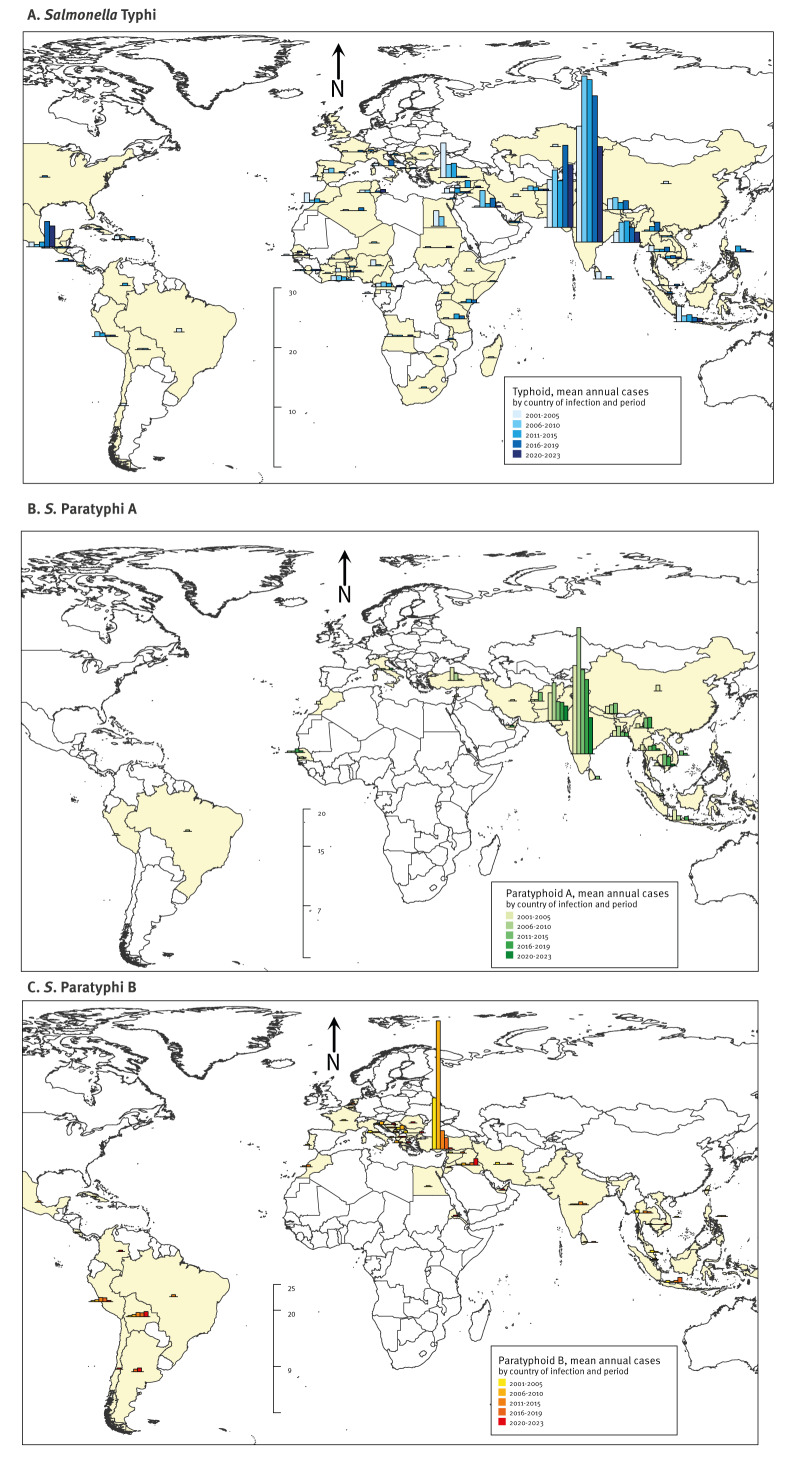
Mean annual imported enteric fever cases with exposure in one country abroad by suspected country of acquisition, serotype and time period for A) *Salmonella* Typhi, B) *S*. Paratyphi A, C) *S.* Paratyphi B, Germany, 2001–2023, (n = 2,133)

Of 2,221 imported cases with exposure on one continent, 84.2% were acquired in Asia (including Türkiye), 6.8% in Africa, 6.2% in the Americas and 2.9% in Europe. Of the imported cases, 97.4% with SPA, 81.9% with STY and 75.1% with SPB infections were exposed in Asia, followed by the Americas and Africa for SPB and STY, respectively (Figure 4, Table 1). The number of imported EF cases exposed in one country abroad by suspected country of acquisition and serotype is shown in Supplement S2.

Countries that were reported at least once as the only country of exposure abroad are coloured in yellow.

Among 2,133 imported cases with one country of exposure, India (38.6%), Pakistan (14.2%) and Türkiye (13.1%) were most commonly reported (Table 1). Persons under 18 years of age comprised 16.9% (139/824; STY: 20.0% (107/534), SPA: 10.5% (28/267)), 47.0% (141/300; STY: 53.6% (119/222), SPA: 27.6% (21/76)) and 61.1% (171/280; SPB: 67.0% (136/203), STY: 25/54, SPA: 6/15) of cases acquired there, respectively.

India and Pakistan were the top two countries of exposure for STY and SPA cases. Mean annual STY importations from Pakistan and Mexico increased since 2016 (Table 1, Supplement S1). Türkiye was the top country of acquisition of SPB. Proportions of autochthonous SPB cases (overall median age 11 years, range 0-92) and importations from Türkiye among cases exposed in a single country abroad were significantly lower in 2013–2023 compared with 2001–2012 (14.9% (15/101) vs 37.8% (157/415), p < 0.0001 and 23.1% (18/78) vs 73.4% (185/252), p < 0.0001). Concurrently, the proportion of SPB cases imported from the Americas (35.3% vs 5.9% 2001–2012) and Iraq increased (Supplement S1, Figure 1D, Figure 4).

Excluding known new entrants or visitors to Germany, the 1,481 cases with information had been abroad for a median of 28 days (range: 1–3,270, interquartile range: 22); 4.7% and 16.7% travelled for 1 week or less and 2 weeks or less, respectively (Table 1 with additional information).

Travel reason was not systematically collected. Of 64.5% of imported cases with dates of onset and travel period information, 95.2% developed symptoms abroad (30.8%) or within 28 days of arrival in Germany (64.4%).

*Salmonella* Paratyphi B cases were significantly more likely to develop symptoms abroad (43.1% vs 28.3% (339/1,199) than cases with other serotypes, p < 0.0001). A lower proportion of SPB cases fell ill more than 10 days after arrival in Germany than SPA cases (8.4% vs 29% p < 0.0001). Onset by serotype is shown in Table 1.

### Estimated incidence among air travellers

The highest overall EF incidences per 100,000 air travellers were estimated for Pakistan, Bangladesh, Myanmar, Cambodia and Bolivia, while incidences increased after 2012 for Senegal, Mexico and Argentina. Estimated incidences are shown in Tables 2 and 3.

**Table 2:** Number of notified enteric fever cases with stay in a single country abroad per 100,000 air travellers with that destination originating from Germany among countries of acquisition reported for ≥ 5 cases overall, by country and time period, Germany 2012–2023

EF total	2012–2015			2016–2019			2020–2023			Ratio (2016–2019/2012–2015)		Ratio (2020–2023/2016–2019)		Ratio (2020–2023/2012–2015)		2012–2023		
	Number of notifcations	Cases/100,000 air travellers	95% CI	Number of notifcations	Cases/100,000 air travellers	95% CI	Number of notifcations	Cases/100,000 air travellers	95% CI							Number of notifcations	Cases/100,000 air travellers	95% CI
Pakistan	41	22.83	16.38–30.97	66	27.79	21.49–35.35	50	20.87	15.49–27.52	1.22	**↑**	0.75	↓	0.91	↓	157	23.91	20.32–27.96
Bangladesh	16	30.23	17.28–49.09	13	20.91	11.13–35.76	7	14.60	5.87–30.09	0.69	↓	0.70	↓	0.48	↓	36	22.08	15.47–30.57
Myanmar	10	15.85	7.60–29.15	12	16.47	8.51–28.77	0	0	0.00–45.14	1.04	**↑**	NA	↓	NA	↓	22	15.27	9.57–23.11
Cambodia	10	22.06	10.58–40.57	7	9.92	3.99–20.44	3	8.53	1.76–24.92	0.45	↓	0.86	↓	0.39	↓	20	13.24	8.09–20.44
Bolivia	2	12.57	1.52–45.40	3	9.89	2.04–28.91	4	11.41	3.11–29.21	0.79	↓	1.15	**↑**	0.91	↓	9	11.07	5.06–21.01
Nepal	11	10.03	5.01–17.94	8	6.86	2.96–13.52	0	0	0.00–7.00	0.68	↓	NA	↓	NA	↓	19	6.81	4.10–10.64
Senegal	1	3.05	0.08–16–98	2	4.13	0.50–14.94	2	4.97	0.60–17.97	1.36	**↑**	1.20	**↑**	1.63	**↑**	5	4.12	1.34–9.61
India	150	4.24	3.59–4.98	146	3.57	3.02–4.20	88	4.07	3.27–5.02	0.84	↓	1.14	**↑**	0.96	↓	384	3.93	3.54–4.34
Afghanistan	4	4.30	1.17–11.00	1	0.96	0.024–5.33	1	2.64	0.07–1.47	0.22	↓	2.76	**↑**	0.62	↓	6	2.55	0.94–5.54
Peru	6	3.31	1.21–7.19	5	2.07	0.67–4.83	2	1.74	0.21–6.28	0.63	↓	0.84	↓	0.53	↓	13	2.41	1.29–4.13
Iraq	3	1.16	0.24–3.38	8	2.19	0.95–4.32	6	1.53	0.56–3.32	1.89	**↑**	0.70	↓	1.32	**↑**	17	1.67	0.97–2.68
Indonesia	9	1.54	0.70–2.92	9	1.18	0.54–2.24	2	0.52	0.06–1.86	0.77	↓	0.44	↓	0.34	↓	20	1.15	0.70–1.78
Philippines	6	1.78	0.65–3.86	2	0.49	0.06–1.77	1	0.42	0.01–2.35	0.28	↓	0.86	↓	0.24	↓	9	0.92	0.42–1.74
Tanzania	4	2.08	0.57–5.32	2	0.74	0.09–2.68	0	0	0.00–1.60	0.36	↓	NA	↓	NA	↓	6	0.87	0.32–1.89
Mexico	4	0.29	0.08–0.74	18	0.97	0.57–1.53	14	1.12	0.61–1.88	3.36	**↑**	1.16	**↑**	3.89	**↑**	36	0.80	0.56–1.11
Sri Lanka	4	0.81	0.22–2.08	0	0	0.00–0.63	1	0.44	0.01–2.42	NA	↓	NA	**↑**	0.54	↓	5	0.38	0.12–0.89
Argentina	0	0	0.00–0.60	2	0.29	0.04–1.06	3	0.81	0.17–2.37	NA	**↑**	2.76	**↑**	NA	**↑**	5	0.30	0.10–0.70
Lebanon	4	0.61	0.17–1.55	1	0.11	0.00–0.60	1	0.13	0.00–0.74	0.18	↓	1.25	**↑**	0.22	↓	6	0.26	0.09–0.56
Vietnam	4	0.58	0.16–1.48	1	0.11	0.00–0.61	0	0	0.00–0.76	0.19	↓	NA	↓	NA	↓	5	0.24	0.08–0.56
Thailand	7	0.24	0.10–0.49	7	0.19	0.08–0.38	2	0.12	0.01–0.44	0.79	↓	0.65	↓	0.51	↓	16	0.19	0.11–0.31
Morocco	4	0.26	0.07–0.66	1	0.04	0.00–0.24	0	0	0.00–0.31	0.17	↓	NA	↓	NA	↓	5	0.10	0.03–0.23
Türkiye^a^	21	0.08	0.05–0.12	10	0.04	0.02–0.07	2	0.01	0.00–0.03	0.51	↓	0.22	↓	0.11	↓	33	0.04	0.03–0.06
Italy	0	0	0.00–0.01	5	0.02	0.01–0.04	0	0	0.00–0.02	NA	**↑**	NA	↓	NA	→	5	0.01	0.00–0.02

CI: confidence interval; EF: enteric fevers; NA: not applicable.

Ratios > 1 are labelled with arrows in blue cells.

^a^ Number of air travellers is an underestimation of number of travellers as a lot of people travel to Türkiye by car.

**Table 3:** Number of notified enteric fever cases with stay in a single country abroad per 100,000 air travellers with that destination originating from Germany among countries of acquisition reported for ≥ 5 cases for *Salmonella* Typhi, *S*. Paratyphi A and *S.* Paratyphi B, by country and time period, Germany 2012–2023

STY	2012–2015			2016–2019			2020–2023			Ratio (2016–2019/2012–2015)	Ratio (2020–2023/2016–2019)		Ratio (2020–2023/2012–2015)		2012–2023			
	Number of notifcations	Cases/100,000 air travellers	95% CI	Number of notifcations	Cases/100,000 air travellers	95% CI	Number of notifcations	Cases/100,000 air travellers	95% CI						Number of notifications		Cases/100,000 air travellers	95% CI
Bangladesh	15	28.35	15.87–46.75	10	16.09	7.71–29.58	7	14.60	5.87–30.09	0.57	↓	0.91	↓	0.52	↓	32	19.63	13.43–27.71
Pakistan	29	16.15	10.81–23.19	55	23.16	17.45–30.14	42	17.53	12.64–23.70	1.43	**↑**	0.76	↓	1.09	**↑**	126	19.19	15.99–22.85
Myanmar	4	6.34	1.73–16.23	6	8.24	3.02–17.93	0	0	0.00–45.14	1.30	**↑**	NA	↓	NA	↓	10	6.94	3.33–12.76
Nepal	6	5.47	2.01–11.90	6	5.15	1.89–11.20	0	0	0.00–7.00	0.94	↓	NA	↓	NA	↓	12	4.30	2.22–7.51
Cambodia	2	4.41	0.53–15.94	2	2.83	0.34–10.24	1	2.84	0.07–15.84	0.64	↓	1.00	→	0.64	↓	5	3.31	1.07–7.72
India	101	2.86	2.33–3.47	98	2.40	1.95–2.92	64	2.96	2.28–3.78	0.84	↓	1.23	**↑**	1.04	**↑**	263	2.69	2.37–3.03
Afghanistan	3	3.22	0.66–9.41	1	0.96	0.02–5.33	1	2.64	0.07–14.72	0.30	↓	2.76	**↑**	0.82	↓	5	2.12	0.69–4.95
Iraq	2	0.77	0.09–2.79	6	1.64	0.60–3.58	1	0.25	0.01–1.42	2.13	**↑**	0.15	↓	0.33	↓	9	0.88	0.41–1.68
Tanzania	4	2.08	0.57–5.32	2	0.74	0.09–2.68	0	0	0.00–1.60	0.36	↓	NA	↓	NA	↓	6	0.87	0.32–1.89
Philippines	5	1.48	0.48–3.45	2	0.49	0.06–1.77	1	0.42	0.01–2.35	0.33	↓	0.86	↓	0.29	↓	8	0.81	0.35–1.60
Mexico	4	0.29	0.08–0.74	17	0.91	0.53–1.46	14	1.12	0.61–1.88	3.17	**↑**	1.23	**↑**	3.89	**↑**	35	0.78	0.54–1.08
Indonesia	6	1.03	0.38–2.23	3	0.39	0.08–1.15	2	0.52	0.06–1.86	0.38	↓	1.31	**↑**	0.50	↓	11	0.63	0.32–1.13
Lebanon	4	0.61	0.17–1.55	1	0.11	0.00–0.60	1	0.13	0.00–0.74	0.18	↓	1.25	**↑**	0.22	↓	6	0.26	0.09–0.56
Thailand	2	0.07	0.01–0.25	3	0.08	0.02–0.23	0	0	0.00–0.22	1.18	**↑**	NA	↓	NA	↓	5	0.06	0.02–0.14
Türkiye^a^	9	0.03	0.02–0.06	1	0.00	0.00–0.02	1	0.00	0.00–0.02	0.12	↓	1.08	**↑**	0.13	↓	11	0.01	0.01–0.03
**SPA**																		
Cambodia	8	17.65	7.62–34.77	5	7.09	2.30–16.53	1	2.84	0.07–15.84	0.40	↓	0.40	↓	0.16	↓	14	9.27	5.07–15.55
Myanmar	6	9.51	3.49–20.70	6	8.24	3.02–17.93	0	0	0.00–45.14	0.87	↓	NA	↓	NA	↓	12	8.33	4.30–14.55
Pakistan	12	6.68	3.45–11.67	10	4.21	2.02–7.74	8	3.34	1.44–6.58	0.63	↓	0.79	↓	0.50	↓	30	4.57	3.08–6.52
Nepal	5	4.56	1.48–10.64	0	0	0.00–3.16	0	0	0.00–7.00	NA	↓	NA	↓	NA	↓	5	1.79	0.58–4.18
India	43	1.22	0.88–1.64	41	1.00	0.72–1.36	20	0.93	0.57–1.43	0.83	↓	0.92	↓	0.76	↓	104	1.06	0.87–1.29
Thailand	3	0.10	0.02–0.30	3	0.08	0.02–0.23	1	0.06	0.00–0.33	0.79	↓	0.75	↓	0.59	↓	7	0.08	0.03–0.17
**SPB**																		
Bolivia	2	12.57	1.52–45.4	3	9.89	2.04–28.91	4	11.41	3.11–29.21	0.79	↓	1.15	**↑**	0.91	↓	9	11.07	5.06–21.01
Peru	4	2.20	0.60–5.64	3	1.24	0.26–3.63	1	0.87	0.02–4.84	0.56	↓	0.70	↓	0.39	↓	8	1.49	0.64–2.93
Iraq	1	0.39	0.01–2.15	2	0.55	0.07–1.98	5	1.27	0.41–2.97	1.42	**↑**	2.32	**↑**	3.30	**↑**	8	0.79	0.34–1.55
Indonesia	2	0.34	0.04–1.23	4	0.52	0.14–1.34	0	0	0.00–0.95	1.53	**↑**	NA	↓	NA	↓	6	0.35	0.13–0.75
Argentina	0	0	0.00–0.60	2	0.29	0.04–1.06	3	0.81	0.17–2.37	NA	**↑**	2.76	**↑**	NA	**↑**	5	0.30	0.10–0.70
Türkiye^a^	12	0.04	0.02–0.08	9	0.04	0.02–0.07	1	0.00	0.00–0.02	0.80	↓	0.12	↓	0.10	↓	22	0.03	0.02–0.04

CI: confidence interval; NA: not applicable; SPA: *Salmonella* Paratyphi A; SPB: *S.* Paratyphi B; STY: *S.* Typhi.

Ratios > 1 are labelled with arrows in blue cells.

^a^ Number of air travellers is an underestimation of number of travellers as a lot of people travel to Türkiye by car.

### Autochthonous or international outbreaks

During a local autochthonous STY outbreak in 2004 with six cases among members of a pony club, consumption of sandwiches with herb dressing from a local kebab shop was associated with increased risk of disease in a cohort study, although the ultimate source of contamination could not be identified [13].

In 2004, nine cases pertained to an autochthonous SPB outbreak in southern Germany. The source was a local kebab shop with three food handlers asymptomatically excreting SPB of the same phage type and pulsed-field gel electrophoresis (PFGE) pattern as the cases [14].

In 2005, STY transmission occurred in a restaurant employing a cook recently returned from India. Two family members and four restaurant guests subsequently developed autochthonous typhoid fever. All available isolates revealed an identical PFGE pattern [15].

In 2017, three imported STY cases belonged to an international outbreak related to the European Rainbow Gathering held in Italy [16], where thousands of people from around the world camped in a remote area without appropriate sanitary facilities.

In 2023, five autochthonous and genetically related STY cases occurred in two neighbouring districts in Southern Germany. Although a common food exposure was suspected, the source could not be identified.

### Antimicrobial resistance 2017–2023

At least one antibiotic susceptibility test result was reported for 17.5% (102/583) of EF cases since 2017: STY: 18.7% (73/390), SPA: 21.6% (21/102), SPB: 7.4% (5/68).

Ciprofloxacin resistance was reported for 50/59, 16/18 and 1/5 of STY, SPA and SPB cases with information, respectively. Cefotaxime resistance was reported for none of the SPA or SPB cases and 10/56 of STY cases (first in 2019), comprising 9/19 and 1/21 of cases imported from Pakistan and India with information, respectively. Resistance against meropenem, imipenem or ertapenem was not reported.

## Discussion

Our study provides insights into the epidemiologic characteristics and trends of notified EF in Germany over the past 23 years.

Enteric fever cases were mainly imported. The top countries of exposure were India and Pakistan for STY and SPA cases and at least in the early part of the studied period Türkiye for SPB cases. *Salmonella* Paratyphi C cases were rare.

Age and sex distribution of cases in Germany were comparable to reports from other countries [17-19]. Persons under 18 years of age, while only constituting around 17% of the German population, constituted 30% of cases and were overrepresented among autochthonous cases (all serotypes) and imported STY and SPB cases, especially from Türkiye and Pakistan.

The most commonly diagnosed serotype was STY, mainly affecting unvaccinated individuals. The vaccination coverage of only 7% among the cases was comparable to observations in France 2004–2009 (7%), Australia 2010–2011 (7%), US 2008–2012 (6%) and a GeoSentinel analysis 2007–2018 (10%) [20-23], but lower than in the Netherlands 1997–2014 (19%) [24].

Except for during the COVID-19 pandemic travel restrictions, STY notifications remained on a similar level throughout the study period, with higher mean annual importations from Pakistan and Mexico since 2016. We did not observe rising proportions of SPA infections, contrary to some other countries [21,25].

The proportion of SPB infections in our study exceeded findings elsewhere [17,19,20,26-28], even after a marked decrease in the second half of the study period, especially of autochthonous cases and importations from Türkiye. This is probably due to a reduced infection risk in Türkiye, which is the most prevalent country of origin among the population with migration backgrounds in Germany [29].

Investigations into increased numbers of imported SPB cases from Türkiye in 2007-2009 showed that cases usually had a Turkish migration background and had travelled to visit friends and relatives (VFR) during the summer holidays [30-32]. This possibly indicates locally increased endemicity at the time, although Turkish authorities were unaware of outbreaks [30]. Residual immunity of adults and different hygiene behaviours may have contributed to the predominance of persons under 18 years of age amongst imported cases from Türkiye. Also, the age spectrum of travellers to Türkiye may have included more younger children than long-distance travel e.g. to South Asia. During 2013–2023, in line with British surveillance data from 2017 to 2019, a higher proportion of SPB infections was imported from Iraq and South America [17,33]. South Asia being the predominant region for acquiring STY and SPA infections is consistent with reports from most non-endemic countries [17,19,22,24,26-28,34-36]. In contrast, 53% of EF cases in France in 2004–2009 were imported from Africa [20], probably due to a higher proportion of the French population having ties to Africa than to South Asia.

The observed seasonality of cases is in line with European EF data and is probably related to travel patterns [19]. As in French surveillance data [20], peaks of imported cases were not clearly followed by peaks of secondary autochthonous cases, indicating that transmission from imported cases was low.

While the median travel duration was 4 weeks, EF was also acquired by short-term travellers, with 17% travelling for 2 weeks or less. This is comparable to recent GeoSentinel findings 2007–2018 (19%) [23].

Although reason for travel was not reported, median travel duration and information from German outbreaks and other countries, suggest a substantial proportion of cases was likely travelling to VFR [18,21,22,28,35,37]. Travellers VFR are a recognised high-risk group for EF as they are less likely to seek pre-travel health advice than other travellers and may have a higher risk of exposure due to longer travel durations and an increased likelihood of adopting local practices regarding food and water consumption [22,38-41]. Reasons for low uptake of pre-travel consultations - ideal opportunities for vaccination - may be lack of risk awareness, travel at short notice and language and financial barriers [23]. A 2020 online survey among US immigrants originally from South Asia showed almost 80% would consider typhoid vaccination [42], highlighting the need for alternative ways to reach at-risk travellers not attending travel clinics with vaccination offers and hygiene advice. Routine health visits could be used to inquire about future travel plans with provision of vaccinations. In addition, travel operators could link to sites providing information addressing risks and measures in different languages.

The majority of imported EF cases developed symptoms within 28 days of arrival in Germany, consistent with other studies [20,43]. Like the British surveillance data from 2007 to 2011 and Israeli outbreak data from 2009, our data suggest that the incubation period of SP, especially SPA, infections may exceed the maximum of 10 days commonly quoted in the literature, and suggest a shorter incubation period for SPB than SPA [43,44].

Fever was present in almost all cases, and as in other non-endemic countries, diarrhoea was the most commonly reported gastrointestinal symptom [37].

More than 75% of cases were hospitalised, which is similar to reports from the US for 2008–2012 (STY: 77%, SPA: 69%) [21], surpassing 59% in a GeoSentinel analysis 2007–2018 [23] but slightly lower than observations in other European countries [17,20,36]. This is possibly at least partly due to a higher proportion of SPB cases in Germany, which according to our data, were less likely to be hospitalised. Frequent admission has previously been ascribed to the need of timely investigation of febrile illness and administration of intravenous antibiotics.

As in other non-endemic countries, the case fatality rate was less than 1% [18,21,27,28,35]. However, post-mortem diagnosis in three of four fatal cases suggests difficulties in diagnosing EF and therefore we also expect a degree of underdiagnosis of non-fatal cases. The proportion of non-diagnosed cases with only mild or non-specific symptoms may differ by serotype.

In addition, diagnostic sensitivity is limited [34,45] and further reduced by pre-diagnostic administration of antibiotics. During the autochthonous STY outbreak in Germany in 2004, three of six cases received pre-diagnostic antibiotics [46]. In two tertiary hospitals in Spain in 2000–2014, 67% of autochthonous and 60% of imported EF cases had received empiric antibiotics before diagnosis [28]. Diagnosis was delayed in 80% of children in a case series in two Belgian teaching hospitals in 2005–2020 [47]. Children with EF in a Canadian tertiary centre in 1985–2013 had visited a median of three physicians and only by day 11 after presentation did 90% receive appropriate antibiotics [37]. Similarly, most children with EF in an Australian study during 2003–2015 had more than one healthcare visit and a median symptom duration of 9 days before diagnosis [35]. Overall, these studies suggest that EF may often go unrecognised, leading to delays in diagnosis and treatment, which might be associated with poorer prognosis.

In our study, ciprofloxacin resistance was reported for the majority of STY and SPA cases with information. While cefotaxime (a proxy for ceftriaxone) resistance was not recorded for SPA and SPB infections, it was documented for almost 18% of STY cases, including nine of 19 cases with respective information that were imported from Pakistan and only one case exposed elsewhere (India). Data on antimicrobial susceptibility was voluntarily provided within the framework of mandatory notification of Enterobacterales with carbapenem-nonsusceptibility. Therefore, such data have only been available since 2017, are not tailored to EF and classification of these data into multidrug-resistant (MDR) or XDR-STY is not possible. Antimicrobial data were missing for the majority of cases, likely resulting in a notification bias.

In comparison, 72.5% (111/153) and 5.5% (8/145) of STY isolates and 49/52 and 2/51 of SPA isolates with submitted information to the voluntary laboratory-based antibiotic resistance surveillance in Germany during 2017–2023 were resistant to ciprofloxacin and cefotaxime, respectively (personal communication, Felix Reichert, 23 January 2025). If more than one isolate was submitted for an individual, the query included only the first isolate per year. In addition, 67.3% (202/300) and 9.3% (28/300) of STY isolates and 90% (108/120) and 0 (0/120) SPA isolates tested at the NRC during the same period were resistant to ciprofloxacin and ceftriaxone, respectively (unpublished data), i.e. lower than notified for STY and slightly higher or similar for SPA. According to 2019 European surveillance data, 71% of STY isolates were resistant to ciprofloxacin and 13% to cefotaxime [19].

Despite the described limitations, our findings are consistent with high levels of fluoroquinolone resistance detected in other countries [17,35] and reports of occurrence and now dominant circulation of XDR-STY in Pakistan since 2016 [48]. Extensively drug-resistant STY cases have since been reported in many non-endemic countries, mostly affecting people with a recent stay in Pakistan [49], although autochthonous cases have been reported from China and the US [50,51]. In Germany, the RKI was first informed about two children with an XDR-STY infection after a stay in Pakistan in 2018 [52].

In summary, ciprofloxacin is unsuitable for empirical EF therapy and ceftriaxone resistance should be expected in patients exposed to STY in Pakistan. Thus, several countries recommend azithromycin, carbapenems or a combination (depending on the severity) as empirical therapy for patients with a recent stay in Pakistan [17,26].

A more comprehensive overview of AMR patterns (including MDR or XDR infections) based on data sources with higher coverage and including information on all relevant antibiotics and country of exposure would be valuable to inform empirical treatment decisions of clinicians.

In addition, genomic surveillance of travel-associated cases in non-endemic countries can provide valuable information on regional and international spread of emerging resistant strains, including regions where these data are not routinely available [53,54].

Although rare, local outbreaks occasionally occurred in Germany and were usually related to infected food handlers or, in the absence of an identifiable source case, a common place of food consumption [13-15]. One international STY outbreak also affecting Germany was associated with the European Rainbow Gathering. Shigellosis outbreaks related to similar gatherings have also occurred [55].

Some additional limitations need to be considered. *Salmonella* Paratyphi B data may have been affected by a degree of misclassification of the enteric vs the systemic pathovar, despite our efforts to exclude enteric pathovar cases. This might have partially contributed to the higher autochthonous SPB incidence compared with the other serotypes.

National surveillance does not collect information on chronic carriage.

Denominator data for our calculation of incidence per 100,000 air travellers do not consider travellers with other modes of transport (among the countries considered, at least Türkiye is also reachable by car from Germany) and the numerator only includes cases with one country of exposure abroad. Also, a maximum of two destinations on a flight ticket is recorded [56], underestimating travel to more remote areas, and thereby potentially increasing incidence there.

## Conclusion

Although rare in Germany, EF remain important travel-associated diseases with high hospitalisation rates and concerning AMR. Our data provide valuable clues for public health measures. Long travel duration and data from other non-endemic countries and German outbreaks suggest a substantial proportion of cases were travellers VFR, although short-term travellers were also affected. High levels of ciprofloxacin resistance were reported for STY and SPA cases, while cefotaxime (proxy for ceftriaxone) resistance was primarily notified for STY infections imported from Pakistan. To guide empirical therapy, resistance patterns of all relevant antibiotics should be monitored, and analysed ideally by country of infection. Antimicrobial resistance emphasises the importance of prevention, but we found typhoid fever vaccination was greatly underutilised. Many travellers, especially travellers VFR, may not seek pretravel advice, despite willingness to vaccinate. Therefore, primary health providers (including paediatricians) could utilise routine health visits to proactively inquire about travel plans and reach at-risk travellers with risk information and vaccination offers.

## Supplementary Data

270000 Supplementary Material
