# Supplementary material for: Epidemiological characteristics and trends of notified enteric fevers in Germany, 2001 to 2023
Source: Euro Surveill. 2025 Apr 10;30(14):2400314. doi: 10.2807/1560-7917.ES.2025.30.14.2400314 (PMC11987495; doi:10.2807/1560-7917.ES.2025.30.14.2400314)

This supplementary material is hosted by Eurosurveillance as a supporting information alongside the article [Epidemiological characteristics and trends of notified enteric fevers in Germany, 2001-2023], on behalf of the authors who remain responsible for the accuracy and appropriateness of the content. The same standards for ethics, copyright, attributions and permissions as for the article apply. Supplements are not edited by Eurosurveillance and the journal is not responsible for the maintenance of any links or email addresses provided therein.

Supplement 1: Demographic, epidemiological and clinical information of notified enteric fever with subgroup information for *Salmonella* Typhi, *S*. Paratyphi A and *S*. Paratyphi B and suspected country of acquisition by serotype and time period (2001-2012 and 2013-2023), Germany, 2001-2023

	**2001-2012**								**2013-2023**							
	**Enteric fevers, total (n=1,658)**		***S.* Typhi (n=832)**		***S.* Paratyphi A (n=361)**		***S.* Paratyphi B (n=420)**		**Enteric fevers, total (1,012)**		***S.* Typhi (n=666)**		***S.* Paratyphi A (n=201)**		***S.* Paratyphi B (n=109)**	
	**%**	**n/N[a]**	**%**	**n/N[a]**	**%**	**n/N[a]**	**%**	**n/N[a]**	**%**	**n/N[a]**	**%**	**n/N[a]**	**%**	**n/N[a]**	**%**	**n/N[a]**
Male	55.2	913/1,655	55.3	458/829	60.9	220/361	52.4	220/420	54.1	545/1,008	54.8	363/662	58.7	118/201	45.9	50/109
Female	44.8	742/1,655	44.8	371/829	39.1	141/361	47.6	200/420	45.9	463/1,008	45.2	299/662	41.3	83/201	54.1	59/109
Age <18 years	34.5	571/1,656	30.2	251/830	16.1	58/361	59.3	249/420	22.7	229/1,011	23.0	153/665	12.4	25/201	37.6	41/109
Age 18-39 years	43.2	715/1,656	47.2	392/830	55.7	201/361	25.0	105/420	55.1	557/1,011	55.2	367/665	58.7	118/201	49.5	54/109
Age 40-59 years	16.1	267/1,656	15.9	132/830	23.0	83/361	10.7	45/420	17.3	175/1,011	16.7	111/665	24.4	49/201	6.4	7/109
Age ≥60 years	6.2	103/1,656	6.6	55/830	5.3	19/361	5.0	21/420	5.0	50/1,011	5.1	34/665	4.5	9/201	6.4	7/109
Handling food for work	2.6	41/1.563	2.8	22/780	3.5	12/341	1.5	6/402	5.3	33/620	5.6	23/410	6.4	8/126	1.6	1/64
**Autochthonous cases**	18.6	303/1,631	11.9	97/817	8.4	30/356	37.8	157/415	6.0	59/982	4.6	30/651	5.1	10/195	14.9	15/101
Male[b]	53.1	161/303	51.6	50/97	63.3	19/30	54.1	85/157	52.5	31/59	63.3	19/30	70.0	7/10	26.7	4/15
Age <18 years,	46.5	141/303	33.0	32/97	30.0	9/30	59.9	94/157	37.3	22/59	30 .0	9/30	40.0	4/10	53.3	8/15
Age ≥60 years	13.9	42/303	16.5	16/97	20.0	6/30	8.9	14/157	8.5	5/59	10.0	3/30	0.0	0/10	13.3	2/15
**Imported cases**	81.4	1,328/1,631	88.1	720/817	91.6	326/356	62.2	258/415	94.0	923/982	95.4	621/651	94.9	185/195	85.2	86/101
Male[b]	55.6	736/1,325	55.4	397/717	60.7	198/326	51.6	133/258	54.2	498/919	54.9	339/617	57.8	107/185	46.5	40/86
Age <18 years	31.8	422/1,327	29.9	215/719	15.0	49/326	59.3	153/258	21.3	196/922	22.7	141/620	10.8	20/185	31.4	27/86
Age ≥60 years	4.4	58/1,327	5.3	38/719	3.7	12/326	2.3	6/258	4.7	43/922	4.7	29/620	4.9	9/185	5.8	5/86
Asia,	85.8	1,128/1,315	82.4	585/710	97.2	315/324	82.5	212/257	80.7	741/918	80.2	495/617	97.3	179/184	51.2	44/86
Africa	7.2	94/1,315	11.6	82/710	1.2	4/324	1.6	4/257	6.1	56/918	7.5	46/617	2.2	4/184	2.3	2/86
Americas	3.4	45/1,315	3.5	25/710	0.6	2/324	5.8	15/257	10.1	93/918	9.7	60/617	0.0	0/184	34.9	30/86
Europe	3.4	44/1,315	2.3	16/710	0.6	2/324	9.7	25(/257	2.2	20/918	1.5	9/617	0.5	1/184	10.5	9/86
Multiple continents	0.3	4/1,315	0.3	2/710	0.3	1/324	0.4	1/257	0.9	8/918	1.1	7/617	0.0	0/184	1.2	1/86
Top 1 country of acquisition[c]	India: 37.3	479/1,286	India: 42.6	298/699	India: 55.5	173/312	Türkiye: 73.4	185/252	India: 40.7	345/847	India: 41.4	236/570	India: 54.7	94/172	Türkiye: 23.1	18/78
Top 2 country/ies of acquisition[c]	Türkiye: 19.7	253/1,286	Pakistan: 15.0	105/699	Pakistan: 16.0	50/312	Bolivia: 2.8	7/252	Pakistan: 17.2	146/847	Pakistan: 20.9	119/570	Pakistan: 15.1	26/172	Iraq, Peru: 10.3	8/78
Top 3 country/ies of acquisition[c]	Pakistan: 12.1	156/1,286	Türkiye: 6.4	45/699	Türkiye: 4.8	15/312	Serbia: 2.4	6/252	Mexico: 4.3	36/847	Mexico: 6.1	35/570	Cambodia: 8.1	14/172	Bolivia: 9.0	7/78
**Duration of stay abroad[d]**																
≤ 1 week[d]	4.5	36/795	3.1	13/415	6.9	13/189	4.5	8/179	4.8	33/686	3.6	16/450	5.7	8/141	11.3	8/71
>1-2 weeks[d]	11.7	93/795	12.3	51/415	10.6	20/189	12.3	22/179	12.4	85/686	9.3	42/450	19.9	28/141	11.3	8/71
>2-4 weeks[d]	32.7	260/795	32.8	136/415	30.2	57/189	34.6	62/179	39.5	271/686	41.3	186/450	31.2	44/141	45.1	32/71
>1-2 months[d]	27.9	222/795	25.1	104/415	23.3	44/189	39,1	70/179	20.0	137/686	20.7	93/450	22.0	31/141	15.5	11/71
>2-6 months[d]	20.6	164/795	23.9	99/415	24.9	47/189	9.5	17/179	20.7	142/686	21.8	98/450	19.9	28/141	15.5	11/71
>6 months[d]	2.5	20/795	2.9	12/415	4.2	8/189	0.0	0/179	2.6	18/686	3.3	15/450	1.4	2/141	1.4	1/71
**Symptom onset of imported cases[e]**																
During stay abroad[e]	31.5	228/723	29.7	113/380	26.1	46/176	42.3	66/156	30.1	220/730	28.5	139/488	26.4	39/148	44.9	31/69
>0-10 days after stay abroad[e]	43.3	313/723	41.1	156/380	41.5	73/176	49.4	77/156	44.7	326/730	42.8	209/488	48.7	72/148	46.4	32/69
11-28 days after satay abroad[e]	20.3	147/723	24.0	91/380	24.4	43/176	7.7	12/156	20.4	149/730	23.6	115/488	20.3	30/148	4.4	3/69
29-60 days abroad[e]	4.7	34/723	5.0	19/380	8.0	14/176	0.6	1/156	4.7	34/730	4.9	24/488	4.7	7/148	4.4	3/69
>60 days after stay abroad[e]	0.1	1/723	0.3	1/380	0.0	0/176	0.0	0/156	0.1	1/730	0.2	1/488	0.0	0/148	0.0	0/69
**Clinical information**																
Fever	90.6	1,502/1,658	92.4	769/832	95.3	344/361	83.8	352/420	97.7	989/1,012	97.9	652/666	99.0	199/201	95.4	104/109
Diarrhoea	71.8	1,191/1,658	67.7	563/832	64.0	231/361	84.8	356/420	70.7	715/1,012	69.8	465/666	62.2	125/201	89.0	97/109
Constipation	4.6	76/1,658	6.3	52/832	3.9	14/361	2.1	9/420	6.1	62/1,012	7.2	48/666	4.0	8/201	4.6	5/109
Abdominal pain	29.0	480/1,658	26.8	223/832	26.3	95/361	35.2	148/420	47.2	478/1,012	46.4	309/666	45.3	91/201	52.3	57/109
Cough	6.5	108/1,658	6.9	57/832	9.7	35/361	3.1	13/420	11.0	111/1,012	11.4	76/666	13.9	28/201	4.6	5/109
Headache	27.0	448/1,658	27.8	231/832	34.6	125/361	20.2	85/420	33.3	337/1,012	35.0	233/666	29.9	60/201	26.6	29/109
Hospitalised	73.5	1,206/1,642	80.1	659/823	78.3	281/359	59.0	245/415	85.7	827/965	90.4	575/636	78.3	148/189	78.1	82/105
Deaths due to disease	0.3	4/1,630	0.3	2/811	0.3	1/360	0.2	1/414	0.0	0/1,006	0.0	0/662	0.0	0/200	0.0	0/109

[a] Number of cases with information.

[b] Among cases with information, sex was male or female.

[c] If only exposed in one country abroad.

[d] If information on duration of stay was available, known new entrants or visitors in Germany were excluded.

[e] If information on timing of symptom onset available.

Information on *S.* Paratyphi C and unspecified *S*. Paratyphi is not shown in this table.

Supplement 2: Number of imported enteric fevers cases with exposure in one country abroad (n=2,133) by suspected country of acquisition and serotype, Germany, 2001-2023

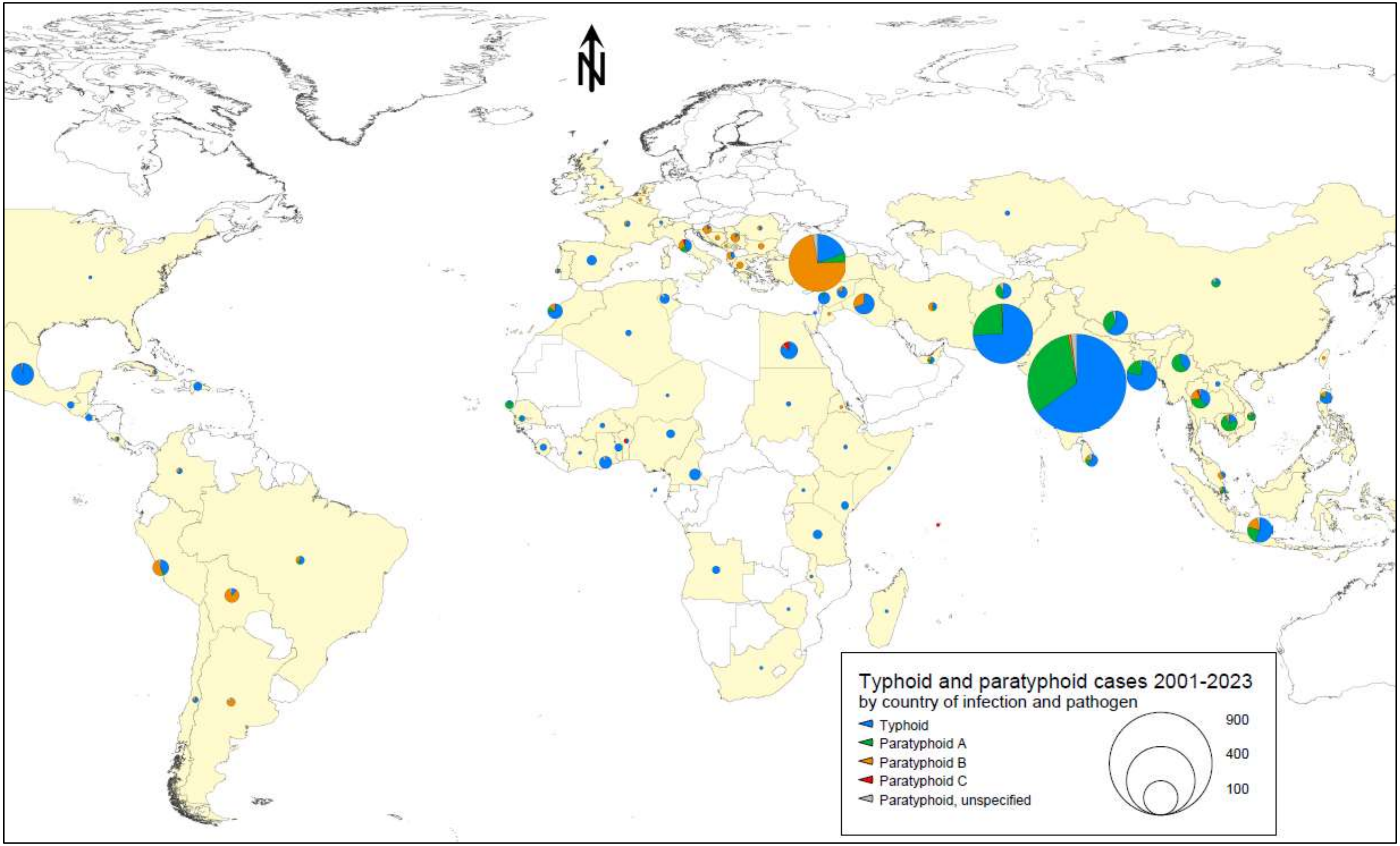

Supplement: Supplementary Material [file 24-00314_ENKELMANN_Supplement.pdf]
